# Supplementary material for: White matter microstructural plasticity associated with educational intervention in reading disability
Source: Imaging Neurosci (Camb). 2024 Mar 14;2:imag-2-00108. doi: 10.1162/imag_a_00108 (PMC11225775; doi:10.1162/imag_a_00108)
Supplement: Supplementary Material [file imag_a_00108-supp.pdf]

Supplementary Materials

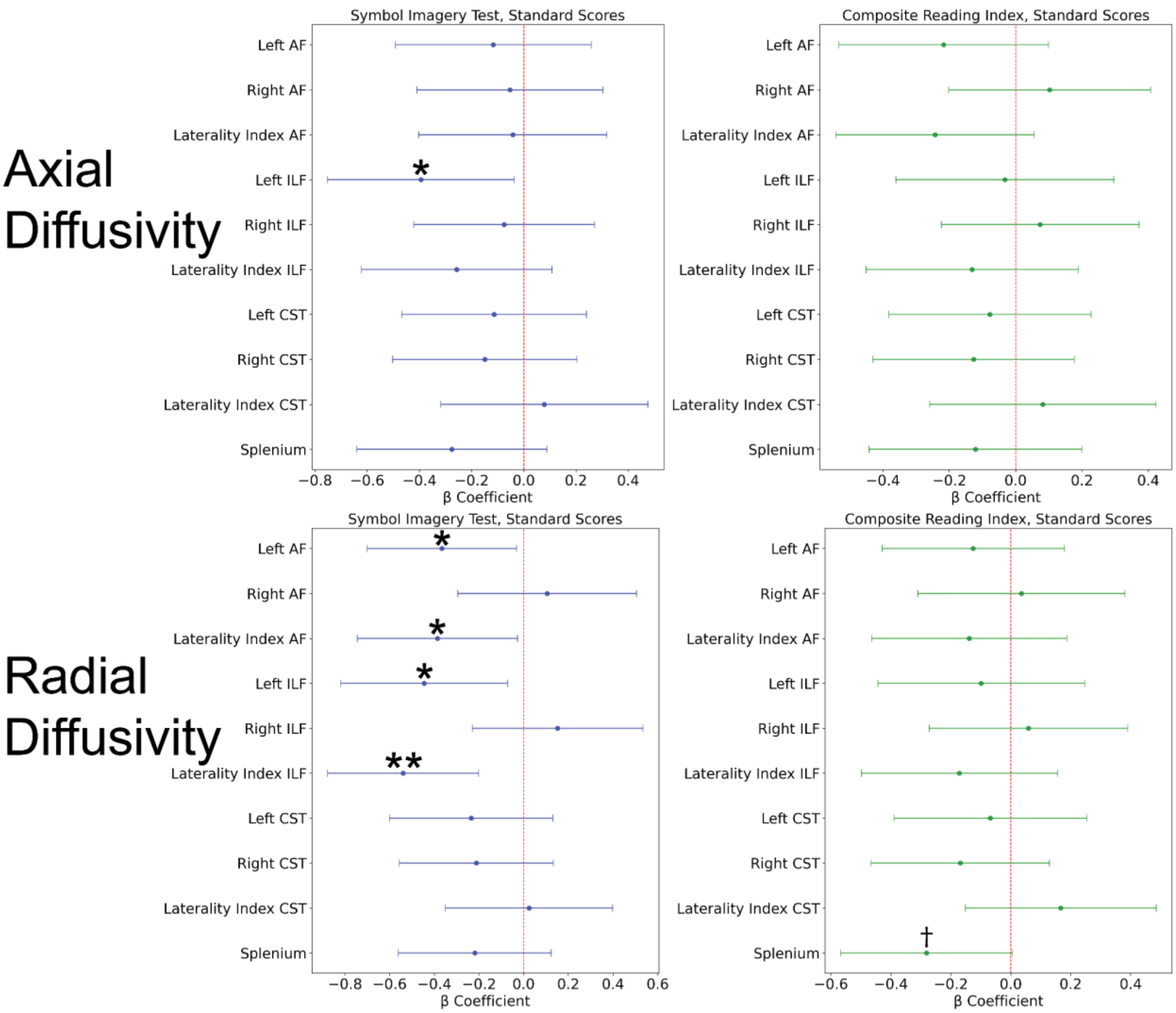

*Figure S1: β coefficient plots for each model using axial diffusivity (top) and radial diffusivity (bottom). β coefficients quantify the relationship between the reading predictor of interest and the microstructural metric, after accounting for nuisance regressors in the models (age, sex, motion index). Error bars represent the 95% confidence interval surrounding the coefficient. †: $p < 0.1$, *: $p < 0.05$, **: $p_{FDR} < 0.05$. Abbreviations: AF - Arcuate Fasciculus; ILF - Inferior Longitudinal Fasciculus; CST - Corticospinal Tract.*

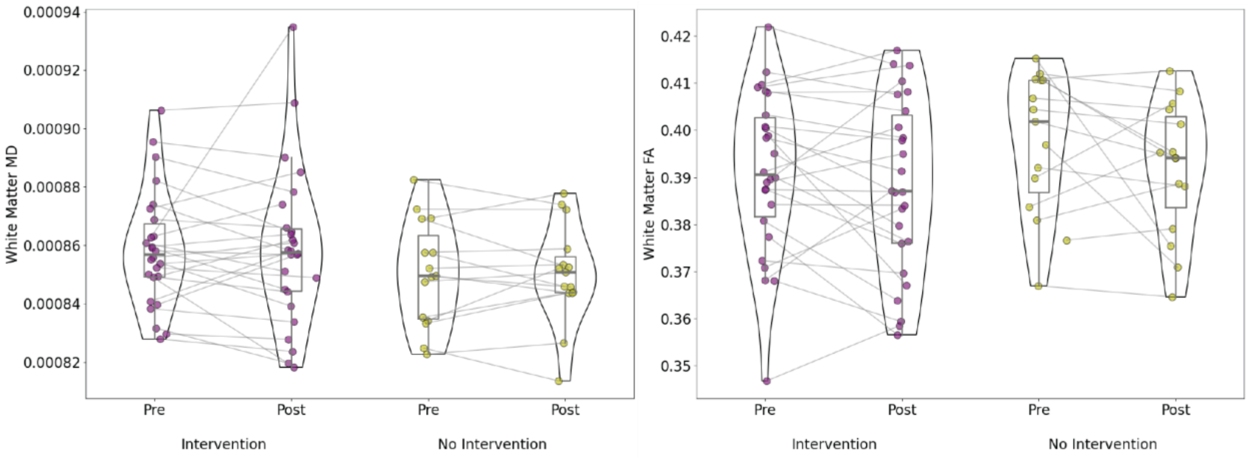

Figure S2: Changes in white matter average mean diffusivity (MD; left) and Fractional Anisotropy (FA; right) for intervention (purple) and non-intervention (yellow) participants. Paired t-tests were used to compare pre and post scores within groups, and two-sample t-tests were used to compare scores at a given time point across groups. No comparisons were statistically significant at a threshold of $p < 0.05$.

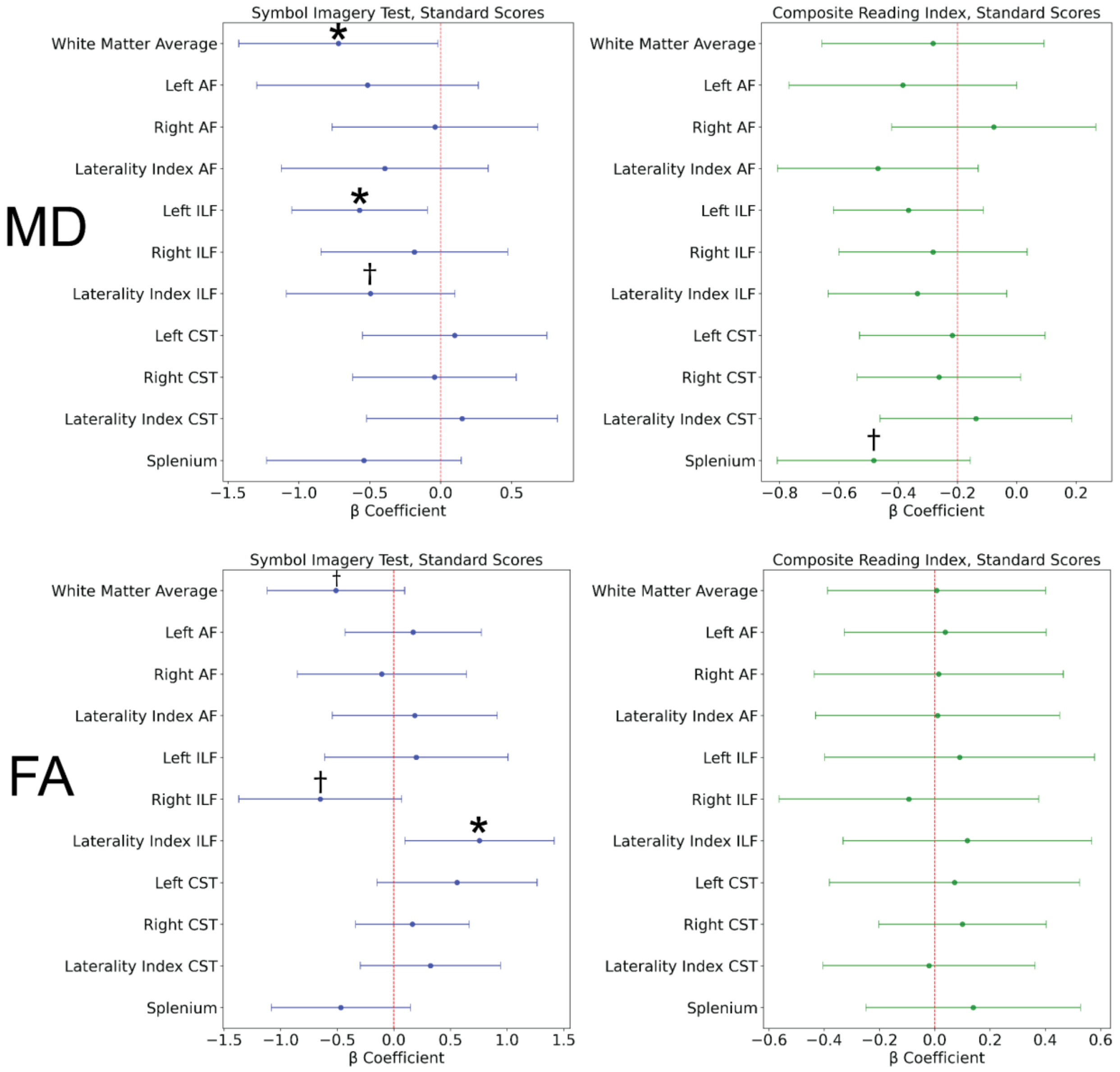

*Figure S3: β coefficient plots for each model run only on the participants who completed the reading intervention (n = 26). β coefficients quantify the relationship between the reading predictor of interest and the microstructural metric, after accounting for nuisance regressors in the models (age, sex, motion index). Error bars represent the 95% confidence interval surrounding the coefficient. †: $p < 0.1$, *: $p < 0.05$, **: $p_{FDR} < 0.05$. Abbreviations: AF - Arcuate Fasciculus; ILF - Inferior Longitudinal Fasciculus; CST - Corticospinal Tract.*

Cross-Sectional Relationships Between White Matter Microstructure and Reading Scores

We ran models to look for relationships between white matter microstructure and reading performance cross-sectionally at each time point across all participants (*Figure*

S4). Nuisance regressors included sex, age, and motion index specific to the time point. Across all participants at the beginning of the summer, decreased leftward ILF MD laterality ($p < 0.05$, $\Delta R^2_{adj} = 0.085$), and both lower MD ($p < 0.1$, $\Delta R^2_{adj} = 0.058$) and higher FA ($p < 0.1$, $\Delta R^2_{adj} = 0.064$) in the left ILF were associated with better SIT scores. Additionally, higher FA in the right ILF ($p < 0.05$, $\Delta R^2_{adj} = 0.106$) and right CST ($p < 0.05$, $\Delta R^2_{adj} = 0.069$), as well as lower MD in the right CST ($p < 0.05$, $\Delta R^2_{adj} = 0.079$), were related to better initial composite reading index scores. At the end of the summer, better composite reading index scores were associated with higher FA ($p < 0.05$, $\Delta R^2_{adj} = 0.084$) and lower MD ($p < 0.05$, $\Delta R^2_{adj} = 0.086$), in the right CST. No tract microstructure values were associated with SIT scores at the end of the summer. Decreasing rightward laterality in right CST MD was associated with better composite reading index scores at the end of the summer ($p = 0.001$, $p_{FDR} < 0.05$; $\Delta R^2_{adj} = 0.209$). This was the only test that survived multiple comparison correction in the cross-sectional analyses.

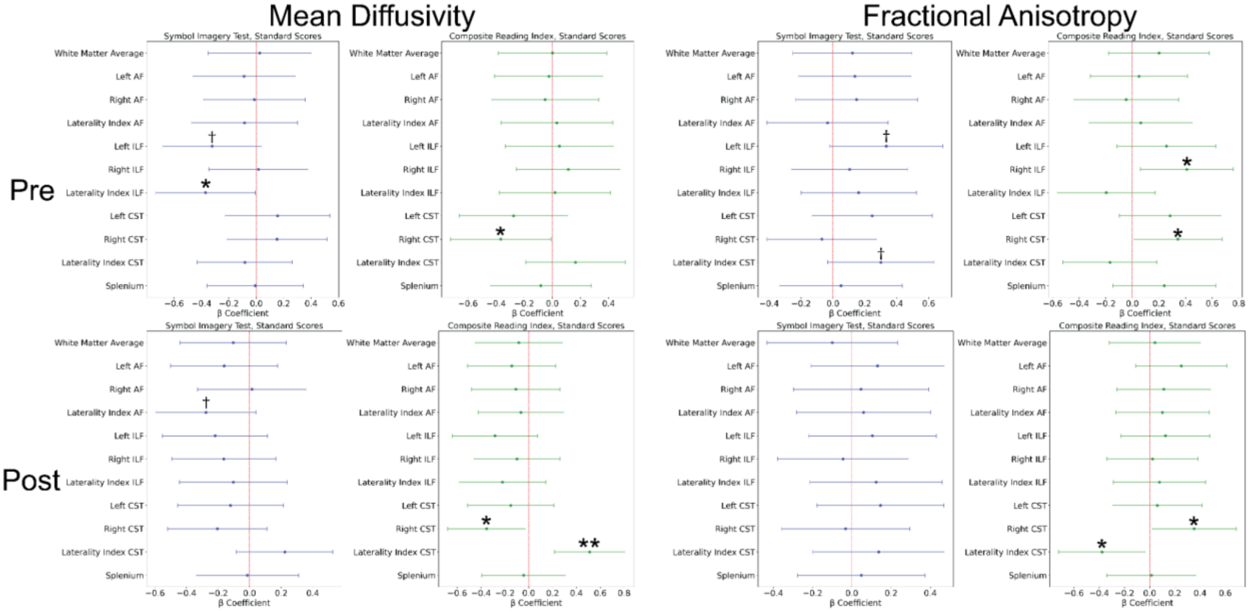

*Figure S4: β coefficient plots for each model in the cross-sectional analyses. β coefficients quantify the relationship between the reading predictor of interest and the microstructural metric, after accounting for nuisance regressors in the models (age, sex, motion index). Error bars represent the 95% confidence interval surrounding the coefficient. †: p < 0.1, *: p < 0.05, **: $p_{FDR} < 0.05$. Abbreviations: AF - Arcuate Fasciculus; ILF - Inferior Longitudinal Fasciculus; CST - Corticospinal Tract.*
